# Antimicrobial effects and mechanisms of hydrogen sulphide against nail pathogens

**DOI:** 10.1038/s41598-025-22062-7

**Published:** 2025-10-31

**Authors:** Fritz Ka-Ho Ho, Alyaa Al-Tabtabai, Sara M. Nasereddin, Osamah S. Malallah, Mark A. Lindsay, Stuart A. Jones, Albert Bolhuis

**Affiliations:** 1 Department of Life Sciences, University of Bath, Claverton Down, Bath, BA2 7AY UK; 2 Faculty of Life Sciences and Medicine, King’s College London, Institute of Pharmaceutical Science, Franklin-Wilkins Building, London, UK; 3 Present Address: College of Pharmacy, Amman Arab University, Mubis, Jordan

**Keywords:** Dermatophyte, Onychomycosis, Nail infection, Hydrogen sulphide, Antifungal, Reactive oxygen species

## Abstract

Nail infections are common but challenging to treat. Oral treatments can be effective but may cause adverse effects and drug interactions, while topical treatments only work in about a third of patients due to poor penetration of antifungals into the nail plate. Small polar molecules, such as hydrogen sulphide (H_2_S), penetrate readily into the nail plate, and previous studies have shown that H_2_S has antimicrobial properties. Using the donor sodium hydrogen sulphide (NaHS), we found that H_2_S has potent activity against causative agents of nail infections, including fungi and bacteria. The most active form appeared to be H_2_S, not the anion HS^−^, but this was most likely related to the faster cellular uptake of H_2_S. We showed that H_2_S inhibits cytochrome C oxidase (COX), a key respiratory enzyme, increases reactive oxygen species and protein *S*-sulfhydration. Transcriptomic analysis revealed a stress response, with 96 genes upregulated and 117 downregulated, indicating efforts to reduce oxidative stress. COX inhibition likely causes electron leakage, generating ROS and oxidising cysteine residues, which then react with H_2_S to form S-sulfhydrated proteins. This novel mechanism, along with the ability of H_2_S to penetrate the nail, suggests topical delivery of an H_2_S donor is a promising new treatment for onychomycosis.

**Supplementary Information:**

The online version contains supplementary material available at 10.1038/s41598-025-22062-7.

## Introduction

Superficial fungal infections are very common, with a global prevalence of 20–25%^1^. This disease, dermatophytosis, can be uncomfortable and painful, reducing the quality of life^2^. In high-risk groups such as the elderly and diabetics, the consequences can be severe. For example, diabetics are 2.7 fold more likely to develop fungal nail infections (onychomycosis) than non-diabetics^3^. In these patients, fungal infections may precede foot ulcers, potentially leading to lower-limb amputation^4^. With rising obesity and type 2 diabetes, more serious cases of dermatophytosis are expected to increase.

The most common cause of dermatophytosis is *Trichophyton rubrum*, though other dermatophytes (*Trichophyton*, *Microsporum* and *Epidermaphyton*) and non-dermatophytes like *Candida*, *Aspergillus*, *Fusarium*, and *(Neo)scytalidium* spp., can also be responsible^5,6^. Finally, bacteria such as *Pseudomonas aeruginosa* or *Staphylococcus aureus* can also infect nails.

Treatment options are limited, mainly involving azoles or allyamines. First-line oral treatments such as itraconazole and terbinafine are reasonably effective, but this takes several weeks or months and may cause side effects and drug interactions. Topical treatments, which are suitable for mild or moderate cases, offer more variety of drugs^7^ and are often preferred by patients. However, they act very slowly and have low cure rates. Nail infections are particularly challenging, often requiring treatment of a year or longer for toenails, with high rates of relapse^8^. Even one of the most effective topical agents, efinaconazole, achieves only a complete cure rate (resolution of clinical signs and symptoms) of 15–18%, and a mycological cure rate (negative for culture or microscopy) of 53–55%^9^.

A key issue is slow penetration of antifungals into the nail plate^10^, influenced by factors such as molecular weight and polarity^10–12^. An “ideal” compound for the treatment should be small, polar, and have good antimicrobial activity. Recently, we identified H_2_S as a promising candidate due to its good nail penetration^13^ and known antimicrobial properties^14,15^. Here we aimed to investigate its activity against microbes causing nail infections and to understand the mechanism of action of this gaseous molecule.

## Materials and methods

### Release of gaseous H_2_S from NaHS

The release of H_2_S gas from 20 mL solutions of 0.1 mM, 1 mM or 10 mM sodium hydrogen sulphide (NaHS) in water was measured in airtight boxes (2.1L; FoodSaver® Quick Marinator). At designated time points, a T101 H_2_S gas analyser (Teledyne) was attached to the box, and the entire box volume was extracted in 4 min at a rate of 650 mL/min for measurement. A separate box was used for each time point/concentration combination. The analyser had a limit of detection (LOD) of 0.4 ppb using a 10 V analogue output range and was calibrated using standard H_2_S gas dilutions provided by the manufacturer.

### Strains and culture conditions

A list of strains is presented in Table 1. Where indicated, isolates were obtained from the American Type Culture Collection (ATCC), the National Collection of Type Cultures (NCTC), the National Collection of Pathogenic Fungi (NCPF), and the Belgian Coordinated Collection of Microorganisms (BCCM). Filamentous fungi were maintained on potato dextrose agar plates that were incubated at 30 °C. Conidia were isolated as described^16^. *Candida albicans* and bacteria were routinely cultured on Brain Heart Infusion (BHI) medium at 37 °C.

**Table 1:** Fungal and bacterial isolates used in this study.

Strain	Notes	Source/reference
*Trichophyton rubrum* ATCC 28,188	Human lesion	ATCC
*Trichophyton rubrum* NCPF 0118	Human nail	NCPF
*Trichophyton rubrum* NCPF 0420	Human	NCPF
*Trichophyton rubrum* NCPF 0719	Human skin	NCPF
*Trichophyton rubrum* NCPF 0936	Human	NCFP
*Trichophyton interdigitale* ATCC 9533	Human	ATCC
*Trichophyton mentagrophytes* NCPF 0748	Dog	NCPF
*Trichophyton indotineae* IHEM 28,378	Human foot, terbinafine resistant	BCCM
*Microsporum canis* NCPF 0179	Cat	NCPF
*Neoscytalidium dimidiatum NCPF* 2192	Human toenail	NCPF
*Fusarium oxysporum* NCPF 2674	Human skin	NCPF
*Aspergillus niger* IHEM 26,751	Human toenail	BCCM
*Sporothrix brasiliensis* IHEM 22,009	Human skin	BCCM
*Candida albicans* SC5314	Human clinical isolate	^17^
*Staphylococcus aureus* NCTC 6571	Methicillin-sensitive	NCTC
*Staphylococcus aureus* MRSA 252	Methicillin-resistant	^18^
*Pseudomonas aeruginosa* PAO1	Chloramphenicol-resistant	^19^
*Escherichia coli* BW25113	Parent strain Keio collection	^20^
*Escherichia coli* DH5α	Cloning strain	^21^

### Antimicrobial activity of H_2_S

The antifungal activity of the H_2_S donor NaHS was tested in liquid using RPMI-1640 medium with glucose and MOPS (RPMI-GM)^22^. Where indicated, the pH was adjusted to pH 5, 7, or 8. The medium was inoculated with ~ 10^5^ conidia/mL of *T. rubrum* ATCC 28,188 and incubated at 30 °C. To limit outgassing of H_2_S, the headspace was kept to ~ 20% of the total volume. After 4 days, cultures were inspected and the minimal inhibitory concentration in aqueous conditions (MIC_aq_) was defined as the lowest concentration with no visible growth.

**Fig. 1:**
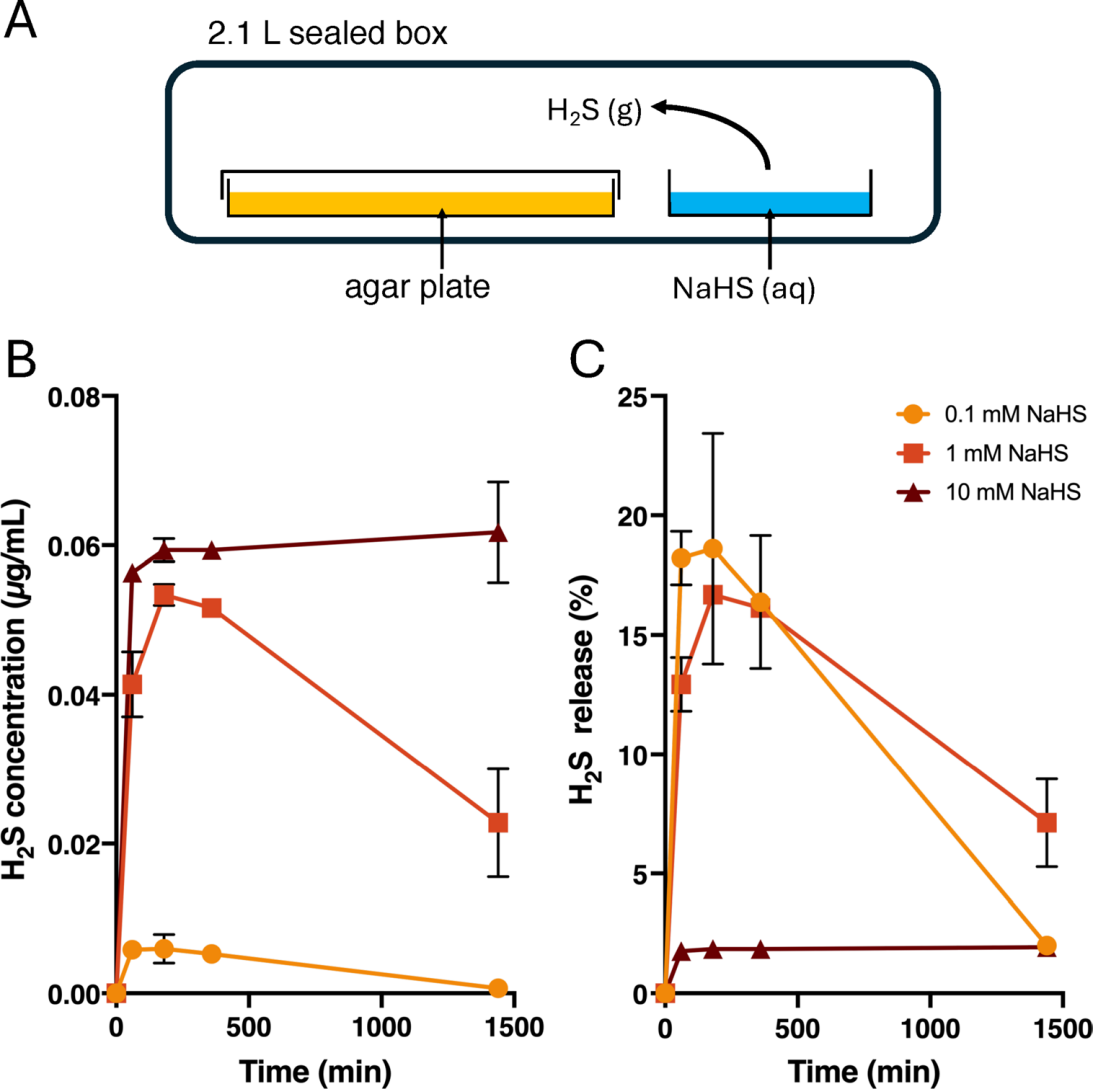
Release of gaseous H_2_S. (**A**) Experimental setup for exposure of microbes to gaseous H_2_S. The gas is released from the donor NaHS, dissolved in 20 mL H_2_O, that is present in an open petri dish that was placed inside a 2.1 L airtight box. (**B**) and (**C**) show the concentration and percentage, respectively, of sulphide released at specific time points in the box. All experiments were repeated independently three times, and the error bars represent the standard deviation.

To test the activity of gaseous H_2_S, conidia of fungi (2 µl of a suspension containing ~ 500 conidia) were inoculated on Sabouraud dextrose agar (SDA) plates, and then the plates (triple vented) were placed in a 2.1 L airtight box containing an open dish with 20 mL of freshly prepared NaHS in water (Fig. 1). Boxes were incubated for 4 days at 30 °C, after which fungal growth was assessed. The MIC_g_max_ was defined as the lowest concentration (assuming that 100% of sulphide is released as gaseous H_2_S) with no visible fungal growth. Plates were then incubated for an additional 3 days at 30 °C without H_2_S: the lowest concentration showing no growth was defined as the MFCg__max_.

To assess the activity of gaseous H_2_S against bacteria and *C. albicans*, overnight cultures were serially diluted in phosphate-buffered saline (PBS), and 10 µL spots were deposited on BHI agar. These plates were placed in boxes with NaHS solution, and incubated at 37 °C. After 24 h, colony-forming units (CFU) were counted. The MIC was defined as the lowest concentration of H_2_S that fully inhibited growth. Plates were incubated for another 24 h in the absence of H_2_S to determine the MFC or minimal bactericidal concentration (MBC).

### Confocal laser scanning microscopy (CLSM)

To assess viability of *T. rubrum* ATCC 28,188 when exposed to H_2_S, conidia (2.5 × 10^5^ CFU/mL) were grown in RPMI-GM (pH 5) for 24 h, in the presence or absence of a sublethal concentration of NaHS. This was followed by staining with the BacLight stain (Invitrogen) according to manufacturer’s instructions. Images were captured using a Zeiss LSM880 laser scanning confocal microscope equipped with Airyscan technology and Zen 3.5 software.

Oxidative stress induction in *T. rubrum* by NaHS exposure was assessed using 2’,7’-dichlorodihydrofluorescein diacetate (DCHF-DA) staining, following a protocol described previously^23^. *T. rubrum* conidia (2.5 × 10^5^ CFU/mL) were cultured at 30 °C for 12 h in Sabouraud dextrose broth (SDB) to induce germination. Next, the germlings were resuspended in RPMI-GM (pH 5) and treated for 2 h with NaHS. Following treatment, the samples were centrifuged at 11,600 g and washed once with RPMI-GM to remove NaHS, resuspended in RPMI-GM containing 10 µM DCHF-DA and 25 µM Calcofluor white, and incubated for 30 min at 30 °C in the dark. After incubation, the samples were washed once with medium and twice with PBS. Images were captured using a Zeiss LSM880 laser scanning confocal microscope as described above.

### Flow cytometry

Fungal germination of *T. rubrum* under exposure to NaHS was examined using flow cytometry. A suspension of 2.5 × 10^5^ CFU/mL of conidia was grown in RPMI-GM (pH 5) with NaHS for 0, 6, 12 and 18 h. Formaldehyde (final concentration at 10%) was added, followed by staining with 20 μg/mL calcofluor white. The cells were left at 4 °C overnight and washed twice with PBS. Blue fluorescence emitted by 20,000 cells was quantified using a BD FACSAria™ III (Becton Dickinson, San Jose, CA, USA) with an 85 μm nozzle tip. Data were collected using BD FACSDiva software and analysed with FCS express.

### Cytochrome C oxidase (COX) assay

*T. rubrum* germlings were prepared by culturing conidia in SDB in a shaking incubator at 30 °C for 19 h, and protoplasts were generated as described^24^. Protoplasts were separated from cell debris by 1 min of vortexing, filtration through a 40 µm strainer, and centrifugation at 700 g for 10 min at 4 °C. The supernatant was carefully collected and centrifuged at 3000 g for 15 min at 4 °C. The pellet was washed with 0.5 mL PBS and centrifuged at 10,000 g for 30 min at 4 °C, and resuspended in 60 µL of PBS. COX activity, with or without NaHS, was measured using a cytochrome C oxidase activity kit (Thermo Fisher). Statistical significance was assessed using a One-way ANOVA with a Dunnett’s post-hoc test using GraphPad Prism 10.4, with *p* < 0.05 considered significant.

### Thiol modification

5’-IAF labelling was used to assess the ability of H_2_S to interact with thiol groups on proteins, using a previously published method^25^. *T. rubrum* conidia were treated with NaHS in RPMI-GM (pH 5.0) for 3 h, washed with PBS, and ground in liquid nitrogen. 2 mL of lysis buffer was added, the samples were vortexed for 10 min at 4 °C, kept on ice for 30 min, and centrifuged at 13,300 g for 20 min at 4 °C. For fluorescent labelling, 2 µL of 20 mM 5’-IAF was added to a 200 µL sample for 10 min, at room temperature in the dark. Proteins were precipitated by adding 1 mL ice-cold acetone and incubation at −20 °C for 10 min. After centrifugation (13,300 g) for 20 min, pellets were air-dried in the dark for 10 min, resuspended in 20 µL of SDS sample loading buffer, and mixed with 2 µL of 10 × DTT. Samples were denatured at 70 °C for 10 min and separated with SDS-PAGE. 5-IAF-labelled proteins were visualised using a UV transilluminator, using a Biorad GelDoc XR + imaging system, followed by Coomassie Brilliant Blue staining to show total protein. Total protein in each lane of the SDS-PAGE gels was quantified using ImageJ/Fiji software (version 2.14.0/1.54f.).

### Transcriptome analysis

Culture preparation and RNA extraction were performed using a previously described method^26^. *T. rubrum* ACTC 28,188 (1 × 10^6^ conidia/mL) was inoculated in 100 mL RPMI-GM (pH 5.0) and incubated at 30 °C for 96 h with agitation. Mycelium was harvested by filtration and transferred to RPMI-GM (pH 5.0) with or without 0.1 mM NaHS, followed by incubation at 30 °C for 3 h. Samples were stored at –80 °C until RNA extraction. Total RNA was extracted from ~ 100 mg frozen mycelia using the Illustra RNAspin Mini RNA Isolation Kit (GE Healthcare) per the manufacturer’s instructions. RNA quantity and integrity were assessed using Qubit RNA BR and IQ assay kits with a Qubit 4 Fluorometer (Thermo Fisher).

Paired-end 100 bp sequencing was performed by BGI Tech Solutions on a DBN sequencer following the construction of an rRNA depletion library. For each condition (with/without 0.1 mM NaHS), 4 biological replicates were sequenced. FastQ sequencing files were quality checked with FastQC, yielding scores of > 36^27^. Reads were splice-aligned to the *T. rubrum* CBS118892 reference genome (ASM151142v1 in NCBI, GCF_000955945.1 Refseq) using Hisat2^28^, with a mean alignment rate of 54.4%. Differential expression analysis was analysed using DESeq2^29^, using the NCBI ASM151142v1 reference database. *P* values were adjusted for transcriptome-wide false discovery rate (FDR), with an adjusted significance threshold of *q* < 0.05. Genes were classified based on Uniprot (www.uniprot.org) annotation, and GO enrichment analysis was performed using FungiDB^30^, using a Benjamini-adjusted p value of < 0.05 as significant). The RNAseq data are available in NCBI BioProject PRJNA1242198.

## Results

### Release of H_2_S gas from the donor NaHS

The setup of exposure of microbes to H_2_S is shown in Fig. 1A, with microbes grown on agar plates placed inside an airtight 2.1 L box, with 20 mL NaHS solution in an open dish. NaHS dissociates in water into Na^+^ and HS^−^, the latter converting to gaseous H_2_S. To quantify H_2_S release, boxes were connected to a gas analyser, and the release of H_2_S was measured after 1, 3, 6, and 24 h, using a separate box for each measurement. The experiments showed that at all concentrations of the donor, H_2_S release peaked within 3 h (Fig. 1B). At 0.1 or 1 mM NaHS, 16–18% of the total available sulphide was released as H_2_S, compared to only 2% with 10 mM NaHS (Fig. 1C). H_2_S levels declined at 6 and 24 h, probably due to adsorption by the box’s plastic^31^.

### Antimicrobial activity of H_2_S

To assess the antimicrobial activity of H_2_S, we tested aqueous sulphide against *T. rubrum* using a standard macrobroth dilution method in which NaHS was dissolved in RPMI-GM. In water, the sulphide exists as H_2_S, HS^−^ or S^2−^, with their proportions pH-dependent (pKa_1_ = 7.04, pKa_2_ = 11.96)^32^. The activity of NaHS was determined at pH 5, 7, and 8. While not affecting the growth of *T. rubrum* significantly, they altered the ratio of [H_2_S]:[HS^−^] to ~ 100:1, 1:1, and 1:10, respectively, with negligible amounts of S^2−^. Notably, *T. rubrum* was more sensitive at pH 5, showing a 20-fold lower MIC_aq_ than at pH 7 or 8 (Table 2). Considering the H_2_S:HS^−^ ratios, this suggests that H_2_S has higher antifungal activity than HS^−^. To note, concentrations of antimicrobials are traditionally expressed in mass per volume, and MIC values of sulphide (i.e. H_2_S plus HS^−^) are therefore also expressed as equivalent concentrations of H_2_S in µg/mL (Table 2).

**Table 2:** Antifungal activity of H_2_S/HS^−^ against *T. rubrum* ATCC 28,188.

pH	MIC_aq_ (µM)^a^	MIC_aq_ (µg/mL)^b^	MIC_g_max_ (µM)^c^	MIC_g_max_ (µg/mL)^c^
5	250	8.5	4.8	0.16
7	5000	170	4.8	0.16
8	5000	170	2.4	0.082

^a^The values given are the sulphide concentrations that, depending on the pH, are either H_2_S, HS^−^ or a mixture of the two.

^b^The values given are the concentrations that would be achieved if all sulphide converts to H_2_S.

equivalent to all sulphide being converted to H_2_S under the presumption that all sulphide is converted.

^c^The values given are the concentrations that would be achieved if all sulphide converts to H_2_S and 100% of that is released in the gaseous form.

To measure gaseous H_2_S activity, SDA plates were inoculated with *T. rubrum* conidia. Similar to the macrobroth tests, SDA plates were also adjusted to pH 5, 7, or 8. Plates inside a box were incubated for 4 days. Supplementary Fig. S1 (top row) shows an example, with the lowest concentration preventing growth defined as the gaseous MIC. Exact H_2_S levels are hard to work out, as they depend on donor concentration and exposure time. Therefore, MIC values are expressed as the MIC_g_max_: the theoretical MIC if all available sulphide is released as H_2_S. Independent of the pH of the agar plates, the MIC_g_max_ was reached with 0.25–0.5 mM NaHS (in 20 mL in the 2.1 L box) that, if all sulphide is released as gas, corresponds to 2.4–4.8 µM (0.082–0.16 µg/mL) of H_2_S (Table 2). Interestingly, H_2_S_g_ was far more potent than H_2_S_aq_: at pH 5, the MIC_g_max_ was 50-fold lower than the MIC_aq_, while at pH 7 or 8 this was 1000–2000 fold lower. It should be noted that the MIC_g_max_ was twofold lower at pH 8 when compared to pH 5 or 7. However, this represents a difference of one dilution step, which is generally considered not significant in MIC testing.

To evaluate the spectrum of activity of H_2_S, several fungal isolates causing superficial infections were tested. These included *T. rubrum* isolates with different morphotypes^33^ (e.g. granular NCPF 0420 and the others with a downy morphology), a terbinafine-resistant isolate of *T. indotinea*, and various non-dermatophytes. The minimal fungicidal concentration (MFC_g_max_) was also determined by incubating SDA plates that were exposed for 4 days for an additional 3 days in the absence of H_2_S. Supplementary Fig. S1 shows an example, and results are summarised in Table 3. From this, dermatophytes (*Trichophyton* and *Microsporum* spp.) appeared more sensitive to H_2_S than most non-dermatophytes, with MIC_g_max_ values of 0.082–0.16 µg/mL vs. 0.32–1.6 µg/mL, respectively. The MFC_g_max_ values were generally the same or double the MIC_g_max_, indicating that H_2_S is fungicidal.

**Table 3:** Antifungal activity of gaseous H_2_S.

Species	H_2_S MIC_g_max_ (µg/mL)	H_2_S MFC_g_max_ (µg/mL)
*T. rubrum* ATCC 28,188	0.16	0.32
*T. rubrum* NCPF 0420	0.16	0.16
*T. rubrum* NCPF 0719	0.082	0.082
*T. rubrum* NCPF 0936	0.082	0.082
*T. interdigitale* ATCC 9533	0.16	0.32
*T. indotineae* IHEM 28,378	0.16	0.16
*T. mentagrophytes* NCPF 0748	0.16	0.16
*M. canis* NCPF 0179	0.16	0.16
*N. dimidiatum* NCPF 2192	0.32	3.2
*F. oxysporum* NCPF 2674	1.6	> 3.2
*A. niger* IHEM 26,751	0.64	3.2
*S. brasiliensis* IHEM 22,009	0.082	0.16
*C. albicans* SC5314	0.64	> 3.2

Next, activity of gaseous H_2_S was tested on bacteria that can cause nail infections^34^. The data (Table 4) illustrated that two *S. aureus* strains, including multidrug-resistant MRSA252, were sensitive to H_2_S, while *P. aeruginosa* was not inhibited at the highest concentration tested. To test resistance in another Gram-negative species, two laboratory strains of *E. coli* were tested. *E. coli* BW25113 was highly resistant (MIC_g_max_ value > 8.2 µg/mL), but the *recA-* strain DH5α showed partial sensitivity with an MIC_g_max_ of 3.2 µg/mL.

**Table 4:** Antibacterial activity of gaseous H_2_S.

Species	H_2_S MIC_g_max_ (µg/mL)	H_2_S MBC_g_max_ (µg/mL)^a^
*S. aureus* NCTC 6571	0.16	0.16
*S. aureus* MRSA252	0.16	0.16
*P. aeruginosa* PAO1	> 8.2	> 8.2
*E. coli* DH5α	3.2	3.2
*E. coli* BW25113	> 8.2	> 8.2

^a^MBC: minimal bactericidal concentration.

**Fig. 2:**
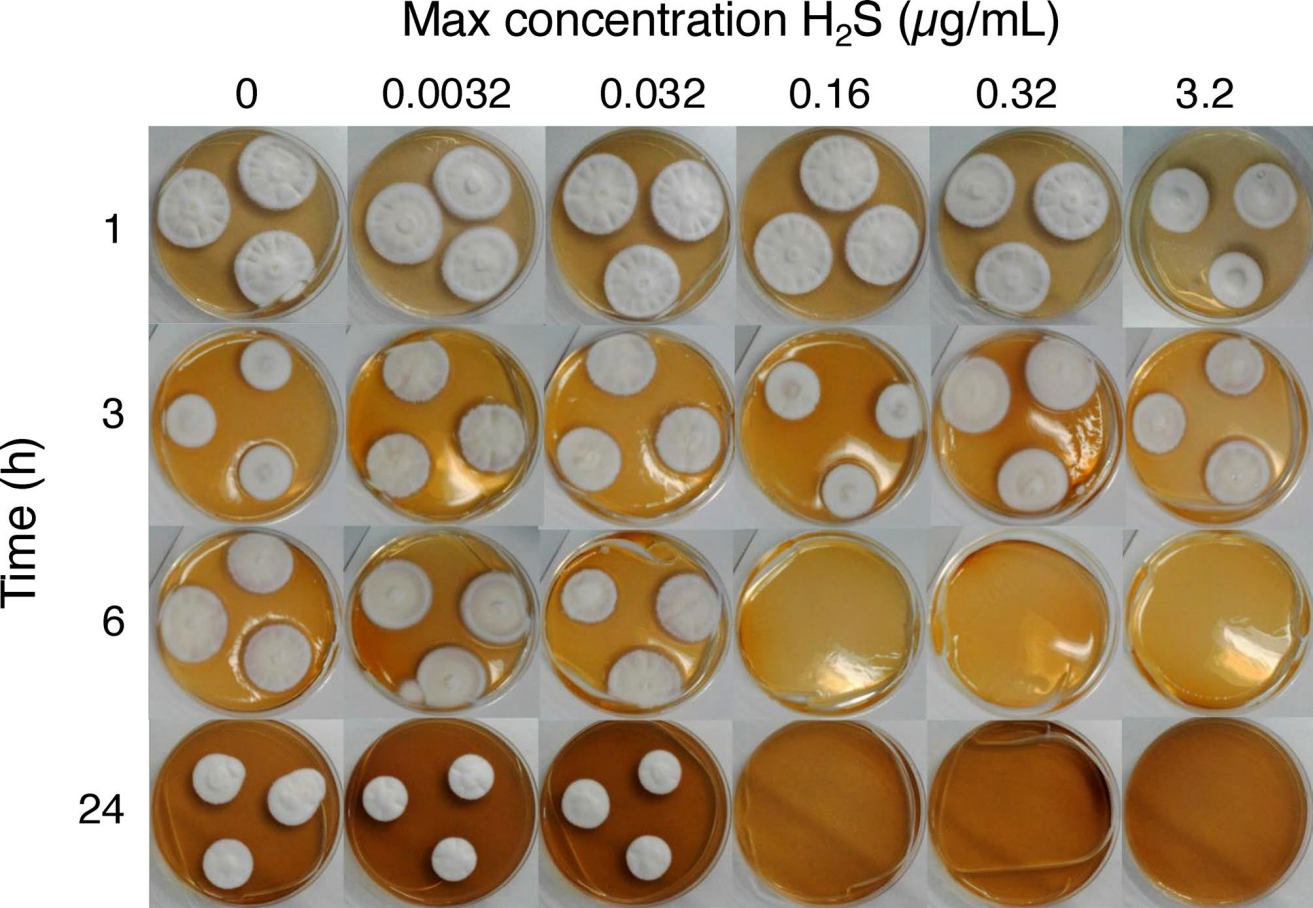
Effect of H_2_S exposure time and concentration against *T. rubrum*. Agar plates were inoculated with 3 spots of *T. rubrum* ATCC 28,188, incubated in airtight boxes for 1, 3, 6, and 24 h, in the presence of different concentrations of H_2_S, followed by a further incubation without H_2_S for a further 7 days. The concentrations H_2_S indicated are those which would be achieved if all available sulphide is released as gaseous H_2_S.

To determine the minimal H_2_S exposure time, SDA agar plates were inoculated with *T. rubrum* and incubated with H_2_S for 1, 3, 6, or 24 h in an airtight box. Plates were then incubated for a further 7 days without H_2_S. This showed that between 3 and 6 h of exposure is sufficient reach the MIC_g_max_ value (Fig. 2).

**Fig. 3:**
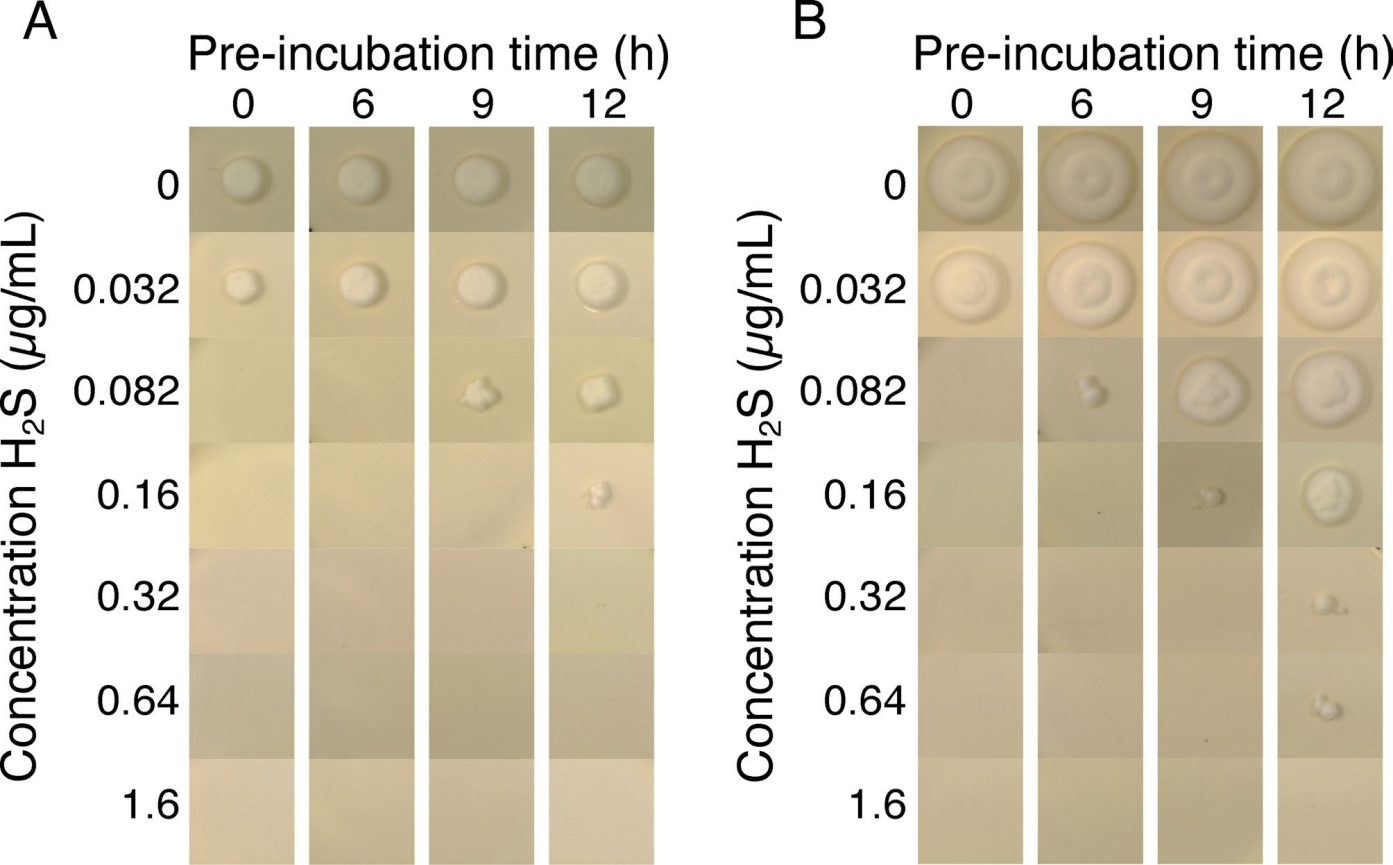
The effect of pre-incubation of *T. rubrum* conidia on the MIC of gaseous H_2_S. *T. rubrum* ATCC 28,188 conidia were deposited on SDA plates and then incubated at 30 °C for 0–12 h, followed by incubation for 4 days in an airtight box containing different concentrations of H_2_S, to determine the MIC_g_max_ (**A**). The plates were removed from the box and incubated for a further 3 days to determine the MFC_g_max_ (**B**).

Germination of *T. rubrum* spores takes several hours, with conidial swelling after 3–4 h and germ tube formation by 9–10 h^16,35^. To test post-germination activity, conidia were pre-incubated to allow germination before H_2_S exposure. The MIC_g_max_ of H_2_S increased after 9–12 h preincubation, suggesting that once germ tubes form, *T. rubrum* becomes more tolerant to H_2_S: the MIC_g_max_ increased from 0.082 to 0.32 µg/mL (Fig. 3A) and the MFC_g_max_ increased from 0.082 to 1.6 µg/mL (Fig. 3B).

**Fig. 4:**
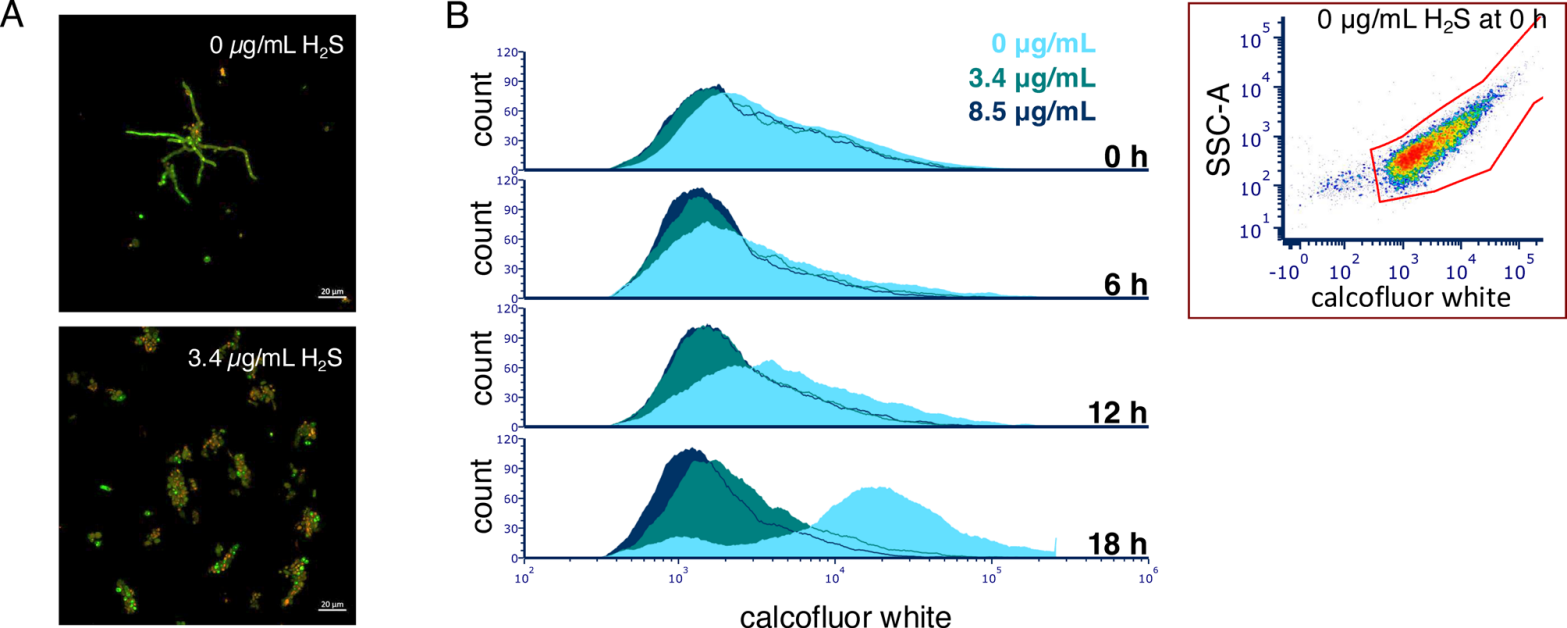
H_2_S inhibits the germination of *T. rubrum* conidia. (**A**) *T. rubrum* ATCC 28,188 without (top panel) or exposed to NaHS in RPMI-GM medium (pH 5) for 24 h, at a sulphide concentration that is equivalent to 3.4 µg/mL of H_2_S. This was followed by staining with the LIVE/DEAD BacLight stain and fluorescence microscopy. (**B**) Flow cytometry analysis of *T. rubrum* ATCC 28,188 conidia exposed to different concentrations of H_2_S in RPMI-GM (pH 5) for 0–18 h. Conidia were identified and delimited on the scatterplot (granularity [SSC-A] vs. blue fluorescence [Calcofluor white; DAPI-A filter]) at the top right corner. Histogram plots show only events from the conidia gate in the scatterplot.

To visualise the effects of H_2_S during germination, *T. rubrum* conidia were exposed to H_2_S in RPMI-GM, stained with Live/Dead Baclight, and examined by confocal microscopy. As shown in Fig. 4A, a sublethal concentration of H_2_S (40% of the MIC_aq_) inhibited mycelium formation (compare top and bottom panels) and resulted conidial clumping, with some conidia staining red, indicating membrane damage. Flow cytometry showed similar results using the fluorophore calcofluor white, which binds fungal cell walls. Without H_2_S, fluorescence increased over time, indicating growth, but with sublethal (3.4 µg/mL) or lethal (8.5 µg/mL) concentrations of H_2_S, fluorescence remained low (compare light blue to teal and dark blue), indicating no conidial outgrowth (Fig. 4B).

### Mechanisms of action of H_2_S

**Fig. 5:**
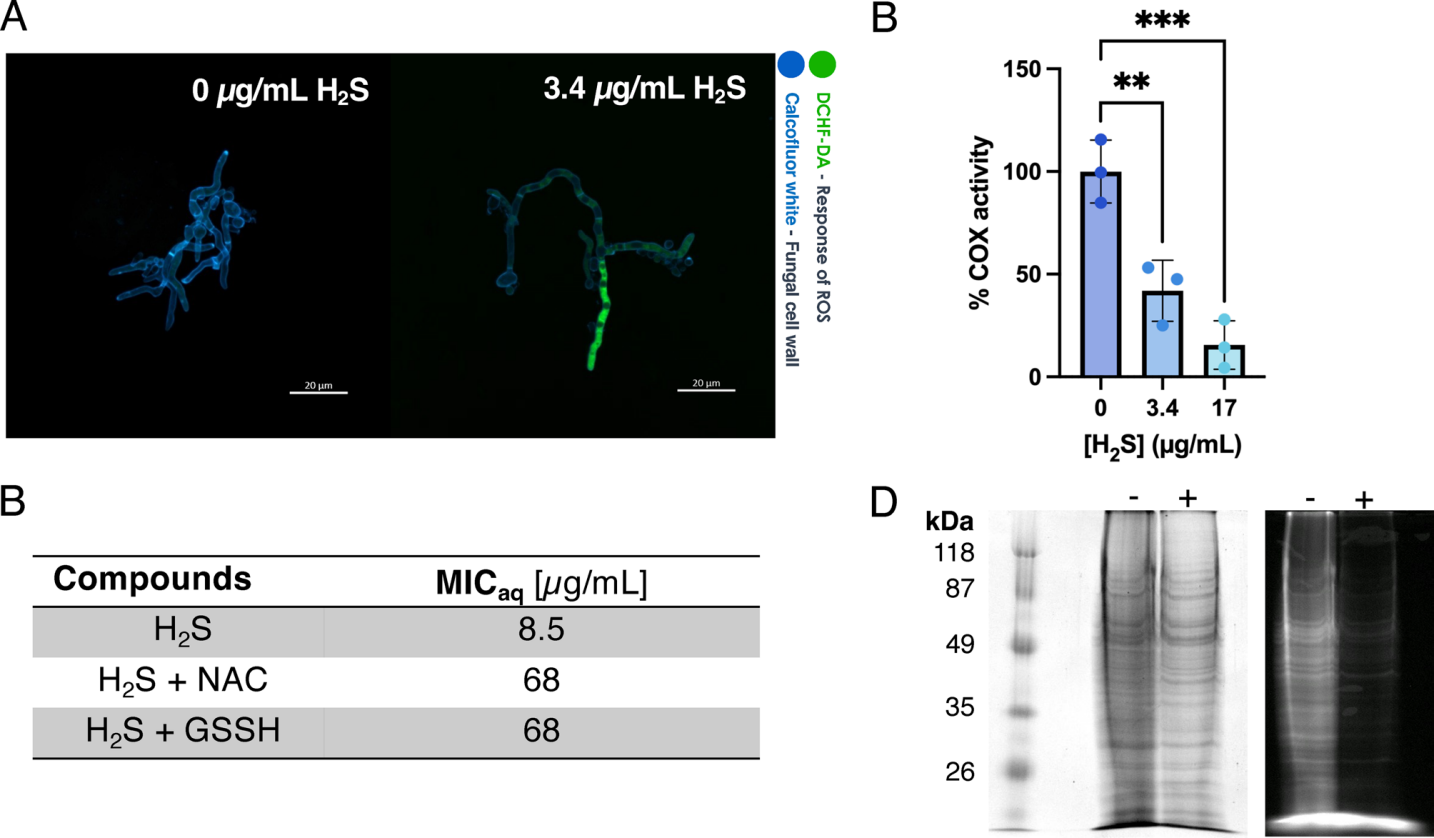
Mechanism of action of H_2_S. (**A**) CLSM images of 12 h pre-incubated *T. rubrum* ATCC 28,188 exposed to NaHS in RPMI-GM medium (pH 5) for 2 h, at a sulphide concentration that is equivalent to 3.4 µg/mL of H_2_S. The samples were stained with Calcofluor white and DCHF-DA. (**B**) 60 µg/mL of the antioxidants N-acetylcysteine (NAC) and reduced glutathione (GSSH) counteract the effect of H_2_S on *T. rubrum*. (**C**) Effect of H_2_S on cytochrome *c* oxidase activity in isolated mitochondria from *T. rubrum*. Statistical analysis was done by a One-way ANOVA followed by Dunnett’s multiple comparison test (**p < 0.01; ***p < 0.001, *n* = 3). (**D**) 5-IAF labelling of proteins from *T. rubrum* conidia, separated by SDS-PAGE. (Left) Coomassie brilliant blue stain of the gel showing molecular weight marker bands (in kDa) and protein extracts from samples that were untreated ( −) or treated with NaHS ( +) for 3 h. (Right) 5-IAF labelled proteins.

Previous studies reported that H_2_S increases reactive oxygen species (ROS) in fungal plant pathogens^14^. Using the redox-sensitive probe DCHF-DA, we investigated ROS levels in *T. rubrum* after 12 h pre-incubation followed by 2 h of treatment with 3.4 µg/mL H_2_S (= 40% MIC_aq_). ROS levels increased compared with the control (Fig. 5A). To confirm this, MIC_aq_ values were determined in the presence of antioxidants. Adding N-acetyl cysteine (NAC) or glutathione indeed significantly increased the tolerance to H_2_S, increasing the MIC_aq_ eightfold (Fig. 5B).

Cytochrome C oxidase (COX), the terminal oxidase in the respiratory chain, is a known cellular target of H_2_S^36^. We tested effects of H_2_S on COX activity in crude mitochondrial extracts of *T. rubrum*. As shown in Fig. 5C, H_2_S inhibited COX activity, with 42% of the activity remaining at 0.4 times the MIC_aq_ (3.4 µg/mL) and 15% at twice the MIC_aq_ (17 µg/mL H_2_S).

H_2_S is also known to induce *S*-sulfhydration, modifying thiol groups in proteins into persulphides (R–S–S–H) ^37^. To assess this, *T. rubrum* was treated with H_2_S and proteins were extracted and labelled with the fluorescent probe 5’-iodoacetamide fluorescein (5-IAF). *S*-sulfhydrated thiols cannot react with 5-IAF, and thus are not labelled. As is shown in Fig. 5D, total protein levels (Coomassie staining, left) were similar between treated and untreated cells. In contrast, 5-IAF labelling revealed many fluorescent proteins in untreated cells, but much less in treated cells. To quantify this, total 5-IAF labelled protein with or without H_2_S was measured by analysing pixel intensity of the SDS_PAGE with ImageJ. To account for differences in protein levels between treated and untreated cells, 5-IAF signals were normalised against Coomassie-stained protein. This showed a 69.5% (+ /−9.7%) reduction in 5-IAF labelled protein when comparing treated against untreated sample (n = 3, *p* = 0.012, paired *t* test). This indicates that H_2_S results in extensive thiol modification.

### Transcriptomic analysis of H_2_S-treated ***T. rubrum***

**Fig. 6:**
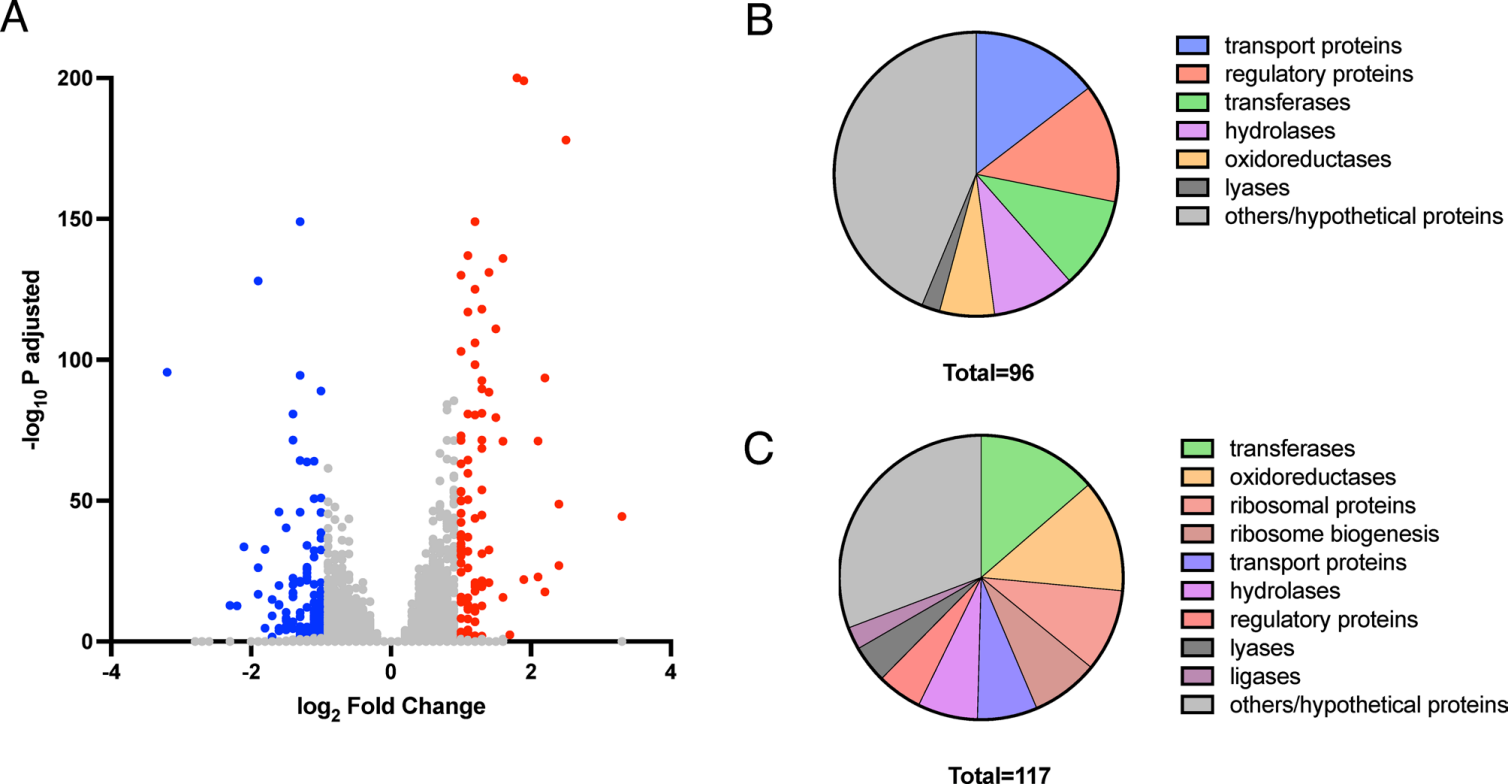
Transcriptome analysis of *T. rubrum* ATCC28188 treated with H_2_S. (**A**) Volcano plot showing differentially expressed genes (absolute log_2_ fold change > 1, adjusted *p* value < 0.05). Red dots represent upregulated genes, blue dots represent downregulated genes, and grey dots indicate genes with no statistically significant differences in their expression. (**B**) and (**C**) show the classification of genes that are upregulated or downregulated, respectively.

Transcriptomic analysis was performed to obtain a more global view of the impact of H_2_S. *T. rubrum* was cultured in RPMI-GM (pH 5) and then treated for 3 h in the absence or presence of a sublethal concentration of NaHS (equivalent to 3.4 µg/mL H_2_S), followed by RNA isolation. After sequencing and data filtering, an average of 24 million high-quality reads of 100-bp paired-end sequences were obtained per sample. The average Q20 and Q30 scores were 97.1% and 91.1%, respectively, confirming high sequence quality. The sequencing files were aligned to the *T. rubrum* CBS118892 reference genome, and differential expression analysis was then performed using DESeq. This revealed that H_2_S resulted in the upregulation of 96 genes and the downregulation of 117 genes. The distribution of the log fold-change and adjusted *p* values (*p* < 0.05) are shown in Fig. 6A. Functional classification of these genes is shown in Fig. 6B and C. A complete list of differentially expressed genes (log_2_-fold change ≥ 1) and their classification is shown in Supplementary Table S1.

**Fig. 7:**
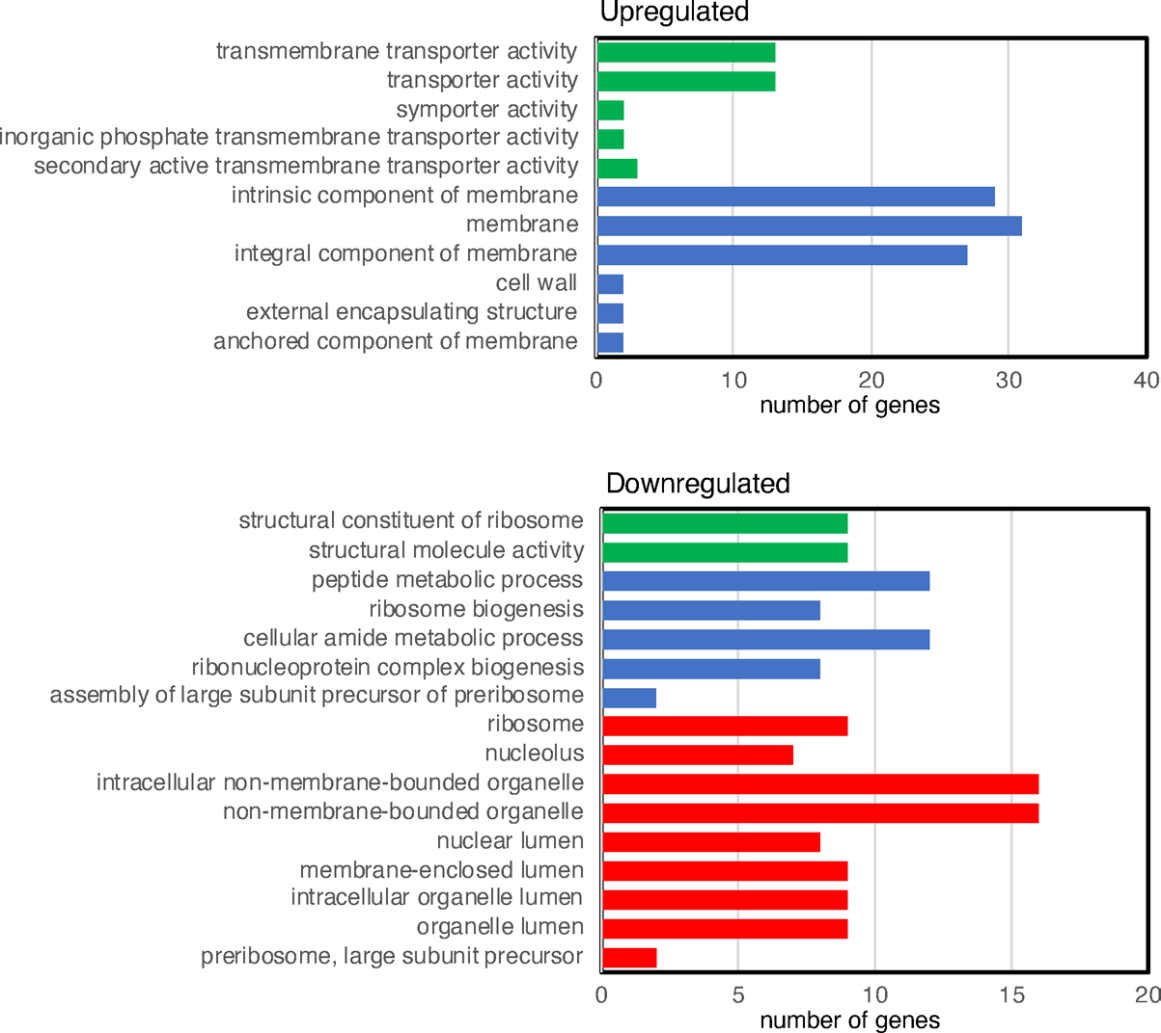
Enrichment analysis of genes that are up- or down-regulated in response to H_2_S. The panels show only GO terms with an adjusted *p* value (Benjamini) of *p* < 0.05. Biological process is shown in green, molecular function in blue, and cellular component in red.

Gene Ontology (GO) enrichment analysis was performed using the FungiDB platform ^30^. As shown in Fig. 7, genes encoding membrane proteins—particularly transporters—were enriched among those that are upregulated. Among downregulated genes, there was enrichment for those encoding ribosomal components or factors involved in ribosome biogenesis.

In the study by Fu et al.^14^, reduced expression of catalase (CAT) and superoxide dismutase (SOD) genes was suggested as a reason for H_2_S-dependent ROS development. The genome of *T. rubrum* contains three putative CAT genes (gene IDs: TERG_01252, TERG_06053, and TERG_02005), and four SOD genes (TERG_07262, TERG_04335, TERG_04819, and TERG_08969). None were downregulated in response to H_2_S in *T. rubrum*, and all but one showed no significant change in expression. The exception was TERG_08969, a gene encoding a putative Cu/Zn SOD, which was upregulated nearly threefold in the presence of H_2_S (Supplementary Table S1).

Due to ROS production in the presence of H_2_S, it was expected that several genes encoding oxidative stress regulators would be differentially expressed. A recent study in *T. rubrum* identified that the transcription factor StuA is involved in regulation of essential antioxidant genes^38^. Several of the genes that were identified in that study showed statistically significant increased expression in response to H_2_S (Table 5), but the levels of overexpression were modest (1.3–1.8-fold).

**Table 5:** Significantly changed expression of genes involved in oxidative stress response.

ID	Description	Log2 fold change
TERG_00714	Cell pattern formation-associated protein (StuA)	0.4
TERG_01117	Stress response regulator SrrA	0.8
TERG_06759	C_2_H_2_ transcription factor	0.6
TERG_07855	Response regulator	0.8
TERG_02940	bZIP transcription factor AP-1/Yap1	0.5

## Discussion

We examined whether exogenous H_2_S has activity against microbes causing nail infections, which are difficult to treat due to poor penetration of antifungals into the nail plate^10–12^. A recent study showed that polar molecules with a low molecular weight (< 120 g/mol) readily penetrate the nail plate. H_2_S, being weakly polar with a molecular weight of 34 g/mol, penetrates nails much more efficiently than topical antifungals such as amorolfine or ciclopirox^13^. Because of its antimicrobial activity, H_2_S was proposed as a promising candidate for onychomycosis treatment, capable of reaching pathogens deep within the nail.

We demonstrated that H_2_S is effective against pathogens causing nail infections, including a terbinafine-resistant isolate of *T. indotineae*. The effects of sulphide differ between gaseous and aqueous forms and, in aqueous conditions, depend on the pH. In solution, there is an equilibrium of H_2_S ⇌ HS^−^ ⇌ S^2−^, with pK_a_ values of 7.04 and 11.96 for the first and second step, respectively. At pH 5, H_2_S predominates, at pH 7 the ratio of H_2_S to HS^−^ is ~ 1:1, and at pH 8, HS^−^ is the major form. The highest activity was observed at pH 5 (a 20-fold lower MIC_aq_ value), suggesting that H_2_S is more active than HS^−^. However, the cytoplasmic pH remains near neutral^39^, and the intracellular ratio of H_2_S:HS^−^ is likely constant. This suggests the differences in activity are primarily related to uptake: H_2_S is less polar than water and can diffuse freely across membranes^40^, whereas the charged HS^−^ ion is likely transported more slowly via proteins. However, membrane permeability is pH-dependent^41^, and because the relative abundance of H_2_S and HS^−^ also varies with pH, it becomes difficult to predict precisely how much of each species crosses the membrane under physiological conditions. For instance, if the external acidic environment was to facilitate the uptake of HS^−^, this could shift the equilibrium towards greater internalisation of the ionised form. Nevertheless, the difference in species activity and the known properties of H_2_S diffusion are the most likely explanations for the observed results.

It should be noted that the pH in the cytoplasm is close to neutral, thus once H_2_S internalises, approximately half of that converts to the anion and the cytoplasmic concentrations of H_2_S and HS^−^ will be similar. This equilibrium makes it difficult to determine which species is primarily responsible for the observed biological effects, and the interconversion between the two further complicates such distinctions. Consequently, in discussions on the antifungal activity of H_2_S, the term also implicitly includes HS^−^.

In the gaseous state, the pH of the growth medium has no effect on H_2_S activity, as in air all sulphide will be in the form of H_2_S which, as mentioned before, freely diffuses into cells. Interestingly, gaseous H_2_S is more active than aqueous H_2_S (at pH 5, the MIC_g_max_ is 50-fold lower than the MIC_aq_). We cannot exclude different modes of action of gaseous and aqueous forms, for instance if some targets are extracellular. However, once H_2_S diffuses into the cytoplasm, the cellular targets are likely to be the same, irrespective of whether H_2_S entered in the gaseous or aqueous form. Therefore, we think that the difference in activity between gaseous and aqueous forms is, similar to what we observed at different pH values, more likely related to the uptake of H_2_S. In the gaseous form, H_2_S can diffuse rapidly across membranes, whereas the aqueous form will interact with water or other solutes through hydrogen bonding^42^, thereby slowing down the rate of diffusion into cells.

It should be noted that the true gaseous MIC values are hard to define in a closed system, as H_2_S release varies with donor concentration and time. For dermatophytes, we found MIC values with 0.25–0.5 mM NaHS in 20 mL, equating to MIC_g_max_ levels of 0.082–0.16 µg/mL. At those concentrations, approximately 16% of sulphide is released as H_2_S after 6 h—time sufficient to kill the fungi—suggesting true gaseous MICs of 0.013–0.026 µg/mL. That compares favourably with topical antifungals such as ciclopirox (0.25 µg/mL) and amorolfine (0.06 µg/mL)^43^. However, these values were determined using different methodology, making a direct comparison difficult. This comparison is also relevant when comparing the MIC_aq_ and MIC_g_max_. Both metrics relate to the total amount of sulphide present. However, in the case of H_2_S gas, the fungi are not exposed to all available sulphide. This indicates that the difference between the aqueous MIC and (true) gaseous MIC is even greater than indicated above. Nevertheless, as the gaseous H_2_S concentration is not constant, a direct comparison between the aqueous and true gaseous MIC is likely unfeasible.

In *T. rubrum*, early stages of growth such as spore germination were more sensitive to H_2_S, as the MIC_g_max_ increased when applied during hyphal formation, which was also observed in plant pathogens^44^. Germination involves rapid metabolic and respiratory changes^45–47^. In *Aspergillus fumigatus*, increased oxygen uptake is observed 3.5 h after germination^46^, while in *Botryodiplodia theobromae*, the activity of COX peaks at 3.5–4 h^47^. H_2_S is known to inhibit COX through binding to the haem moiety, competing with oxygen^48^. Our observation that 3–6 h of H_2_S exposure is needed to inhibit *T. rubrum* conidia aligns with this, and we also identified COX as one of the targets of H_2_S in *T. rubrum*. However, germination is a complex process and other cellular processes may also be affected, since H_2_S can also inhibit other proteins with metal centres^49^.

Similar to previous studies^14,15^, we found that H_2_S increases ROS, based on the response of the redox-sensitive probe DCHF-DA and antioxidant-mediated increases in MIC_aq_ values of H_2_S. Notably, this effect is concentration-dependent; at low endogenous concentrations, H_2_S can function as an antioxidant and a signalling molecule^36^. However, higher concentrations of exogenous H_2_S lead to ROS that potentially damages DNA, lipids and proteins^50^. In this respect, the observation that *E. coli* BW25113 was more resistant than *E. coli* DH5α is interesting: the latter is *recA*^*-*^, a gene involved in DNA repair^51^. Thus, ROS-induced DNA damage may be repaired less efficiently in DH5α, possibly explaining the increased sensitivity of this strain.

Oxidative stress in *A. niger* was linked to H_2_S reducing the expression of genes encoding CAT and SOD^14^, key ROS-scavenging proteins^52^. However, RNA data for *T. rubrum* showed no downregulation of catalase or SOD genes; instead, one was upregulated threefold (see below). A more likely reason for the development of ROS is the inhibition of COX: this results in the dysfunction of the respiratory chain and the leakage of electrons, which in turn contribute to the generation of ROS^53,54^.

We also observed that many proteins are modified through *S*-sulfhydration. This may be a consequence of ROS, as only oxidised cysteines can react with H_2_S^37^. In mammalian cells, endogenous H_2_S mediates signalling via *S*-sulfhydration^55^. In our study, the exogenously applied H_2_S likely resulted in excessive *S*-sulfhydration, thereby impairing protein function^56^.

Transcriptomics revealed many differentially expressed genes, probably reflecting stress responses to H_2_S. One protein likely to be involved in detoxification is a putative Cu/Zn SOD (TERG_88969), the gene of which was upregulated nearly threefold. SODs are required for resistance to oxygen radicals, but a recent study showed that a Cu/Zn SOD (denoted SOD1) also functions as an H_2_S oxidase, protecting against H_2_S toxocity^57^. No catalase gene expression changes were observed, consistent with findings in the yeast *Cryptococcus neoformans*, where catalases were not involved in the oxidative stress response^58^.

We also identified several genes encoding regulatory proteins, including those involved in oxidative stress response. Several of these are predicted to bind Zn^2+^, often coordinated by cysteines^59^. It is conceivable that these are *S*-sulfhydrated which could affect their function, and that *T. rubrum* attempts to compensate for this by upregulating their expression.

Other genes identified included those encoding transport proteins, possibly another response to stress. For example, two phosphate transporters were upregulated (TERG_03172 and TERG_08993), which might be linked to an increased demand for NADPH synthesis. The latter is essential for ROS-detoxifying enzymes such as glutathione and thioredoxin reductases, as was suggested for the phosphate transporter Pho84 in *C. albicans*^60^. An oligopeptide transporter was also upregulated (TERG_07783); it is conceivable that this protein is required for the export of glutathione conjugates to detoxify cells^61^.

Enrichment analysis showed a downregulation of genes encoding ribosomal components and biogenesis factors. This is a common stress response: ribosome biogenesis is an energy-demanding process, and its suppression conserved energy and supports^62,63^.

Finally, it should be noted that the genome of *T. rubrum* is poorly characterised and many affected genes encode proteins with an unknown function.

## Conclusion

We have shown that H_2_S has strong antimicrobial activity and is particularly potent against dermatophytes—including those that are drug resistant. Our findings suggest that H_2_S inhibits COX, leading to leakage of electrons and subsequent ROS generation. H_2_S also causes excessive *S*-sulfhydration of proteins, likely stimulated by ROS. This innovative mechanism of action, combined with the ability of H_2_S to penetrate the nail plate, suggest that topically delivered H_2_S—via donors such as NaHS—is a promising and innovative therapeutic approach.

The potential toxicity of H_2_S must be considered. Previous toxicology assessment suggests that H_2_S does not penetrate human skin^64^ and thus living cell exposure would only occur via open wounds present during any topical administration. In vitro, human cells are relatively tolerant. For example, Caco-2 cells showed a reduced metabolic rate only above 2 mM NaHS and a slightly decreased viability at 3 mM (equivalent to 68 µg/mL H_2_S)^65^. This aligns with reported endogenous serum levels of 20–80 µM, although free H_2_S levels are probably sub-micromolar^66^. Inhaled H_2_S can, however, be toxic. In the UK and the EU, occupational exposure limits are are 5 ppm (8-h time-weighted average) and 10 ppm for short periods (15 min)^67^. However, applying approximately 50 mg of a gel containing 10% of H_2_S (or, more precisely, an equivalent amount of an H_2_S donor) would generate 0.35 ppm if all was released into a small unventilated room (10 m^3^). With the intended topical treatment, actual acute inhalation exposure is expected to be much lower than this theoretical maximum and exposure will therefore remain well below the safety thresholds. Nevertheless, toxicological evaluation will be included in our future studies on the formulation of H_2_S donors.

## Supplementary Information

Below is the link to the electronic supplementary material.

Supplementary Material 1

## Data Availability

RNAseq data are available in NCBI BioProject PRJNA1242198 (https://www.ncbi.nlm.nih.gov/bioproject/PRJNA1242198), and other data are contained within the manuscript.
