# Supplementary material for: Antimicrobial effects and mechanisms of hydrogen sulphide against nail pathogens
Source: Sci Rep. 2025 Oct 31;15:38241. doi: 10.1038/s41598-025-22062-7 (PMC12578836; doi:10.1038/s41598-025-22062-7)
Supplement: Supplementary file 1 — Supplementary Material 1 [file 41598_2025_22062_MOESM1_ESM.pdf]

Supplementary Table S1: Genes that are differentially expressed in the presence of H_2S.

UPREGULATED GENES			
**Transport proteins**			
Gene	Description	Log2FC	Padj
TERG_00008	Major facilitator superfamily (MFS) profile domain-containing protein	1.2	8.72E-107
TERG_00348	Major facilitator superfamily (MFS) profile domain-containing protein	1.2	1.85E-44
TERG_02369	Major facilitator superfamily (MFS) profile domain-containing protein	1.3	1.80E-118
TERG_02819	Amino acid permease	1.1	3.59E-04
TERG_03172	Phosphate transporter	1.2	4.95E-107
TERG_04626	Major facilitator superfamily (MFS) profile domain-containing protein	1.1	1.06E-32
TERG_04766	Vacuolar calcium ion transporter	1.4	3.36E-89
TERG_04952	ABC multidrug transporter MDR5 (Multidrug resistance protein 5)	1.1	4.16E-65
TERG_05141	NCS1 nucleoside transporter	1.1	7.44E-05
TERG_07418	Major facilitator superfamily (MFS) profile domain-containing protein	1	2.86E-46
TERG_07783	Oligopeptide transporter	1.2	2.26E-149
TERG_08104	P-type Na(+) transporter (EC 7.2.2.3)	1.1	2.08E-137
TERG_08993	Phosphate transporter	1.6	3.34E-137
TERG_02101	Amino-acid transporter arg-13	1	2.11E-31
**Regulatory proteins**			
TERG_00724	Zn(2)-C6 fungal-type domain-containing protein	1.2	4.85E-99
TERG_01794	Zn(2)-C6 fungal-type domain-containing protein	1.2	9.94E-08
TERG_03938	Myb-like domain-containing protein	1	2.60E-25
TERG_04032	HTH araC/xylS-type domain-containing protein	1	1.34E-50
TERG_04959	Myb-like domain-containing protein	1.5	2.85E-80
TERG_06891	C6 finger domain transcription factor nscR (Neosartiricin B biosynthesis protein R)	1	1.88E-35
TERG_08434	C2H2-type domain-containing protein	1.3	3.26E-72
TERG_01397	C2H2-type domain-containing protein	1.2	2.07E-11
TERG_06118	C6 transcription factor	1	1.12E-14
TERG_05092	Cell pattern formation-associated protein stuA (Stunted protein A)	1	4.94E-43
TERG_05507	BZIP domain-containing protein	1.3	2.43E-69
TERG_04727	Acetamidase regulatory protein	1.1	9.33E-14
TERG_07681	Velvet domain-containing protein	1.1	1.57E-81

**Transferases**			
TERG_02019	NEK protein kinase	1.1	6.00E-13
TERG_02041	Glutathione S-transferase	1.3	1.82E-90
TERG_00193	Phosphatidate cytidylyltransferase	1	1.75E-28
TERG_02057	Cdp-alcohol phosphatidyltransferase protein	1	7.82E-33
TERG_03943	Phospholipid methyltransferase	1.7	4.2E-03
TERG_01150	Methyltransferase domain-containing protein	2.4	1.45E-49
TERG_02836	Transketolase-like pyrimidine-binding domain-containing protein	1	2.66E-46
TERG_03899	Methyltransferase domain-containing protein	2.1	6.62E-72
TERG_04687	Maltose/galactoside acetyltransferase domain-containing protein	1	1.27E-28
TERG_06396	Acyltransferase 3 domain-containing protein	1.1	8.00E-27
**Lyases**			
TERG_05091	CENP-V/GFA domain-containing protein	1	5.53E-54
TERG_08969	Uncharacterized protein	1.6	7.35E-72
**Oxidoreductases**			
TERG_05518	Short chain dehydrogenase	1	2.07E-34
TERG_08334	Proline dehydrogenase (EC 1.5.5.2)	1	6.96E-131
TERG_04930	Uncharacterized protein	1	5.18E-16
TERG_07239	Enoyl reductase (ER) domain-containing protein	2.5	1.09E-178
TERG_06116	indoleamine 2,3-dioxygenase (EC 1.13.11.52)	1.2	2.69E-125
TERG_03314	TauD/TfdA-like domain-containing protein	1.1	3.48E-16
**Others/hypothetical**			
TERG_01381	AN1-type domain-containing protein	1	3.96E-31
TERG_01787	G-protein coupled receptors family 1 profile domain-containing protein	1.1	8.82E-38
TERG_08533	PAP-associated domain-containing protein	1.2	2.59E-21
TERG_01377	DUF202 domain-containing protein	1	9.45E-74
TERG_00060	BYS1 domain-containing protein	2.2	2.60E-94
TERG_02242	Adhesin	1.5	3.37E-112
TERG_01507	Extracellular membrane protein CFEM domain-containing protein	1	7.41E-54
TERG_08286	Extracellular membrane protein CFEM domain-containing protein	1.3	9.95E-82
TERG_06901	YeeE/YedE family integral membrane protein	2.4	9.31E-28
TERG_03312	RTA1 domain-containing protein	1.1	3.94E-12
TERG_07447	Integral membrane protein	1.3	2.53E-22
TERG_02278	4Fe-4S ferredoxin-type domain-containing protein	3.3	4.05E-45
TERG_03037	Endoplasmic reticulum chaperone BiP (EC 3.6.4.10)	1	6.63E-104
TERG_04471	Rheb small monomeric GTPase RhbA	1.1	2.37E-117

TERG_02431	Exosome complex protein	1	6.11E-09
TERG_06065	Uncharacterized protein	1.3	5.87E-32
TERG_05067	Uncharacterized protein	1.9	7.26E-203
TERG_02086	Uncharacterized protein	1.2	3.63E-81
TERG_11555	Uncharacterized protein	1.2	8.67E-03
TERG_11761	Uncharacterized protein	1	4.67E-02
TERG_11874	Uncharacterized protein	1.2	1.33E-18
TERG_11965	Uncharacterized protein	1.1	4.34E-51
TERG_12218	Uncharacterized protein	1.2	1.41E-21
TERG_12479	Uncharacterized protein	1.3	1.01E-02
TERG_12545	Uncharacterized protein	1.1	5.56E-03
TERG_12618	Uncharacterized protein	1.3	3.31E-20
TERG_12619	Uncharacterized protein	1.6	1.87E-16
TERG_12620	Uncharacterized protein	1	4.20E-04
TERG_00273	Uncharacterized protein	1	1.03E-38
TERG_00327	Uncharacterized protein	1	4.31E-72
TERG_04156	Uncharacterized protein	2.2	2.78E-18
TERG_04160	Uncharacterized protein	1.4	1.21E-21
TERG_04419	Uncharacterized protein	1.3	1.35E-45
TERG_08466	Uncharacterized protein	1.2	8.84E-13
TERG_07798	Uncharacterized protein	1.4	3.51E-33
TERG_07720	Uncharacterized protein	1.1	9.16E-09
TERG_06839	Uncharacterized protein	1.2	2.57E-20
TERG_06851	Uncharacterized protein	2.1	1.26E-23
TERG_05363	Uncharacterized protein	1	1.04E-50
TERG_01012	Uncharacterized protein	1.1	2.20E-60
TERG_01418	Uncharacterized protein	1.3	1.83E-13
TERG_03388	Uncharacterized protein	1.3	1.53E-54
TERG_03526	Uncharacterized protein	1	3.45E-33
**DOWNREGULATED GENES**			
**Ribosome components**			
TERG_00106	60S ribosomal protein L24	-1.2	1.76E-64
TERG_00217	Ubiquitin-40S ribosomal protein S27a	-1	2.07E-39
TERG_01550	60S ribosomal protein L38	-1	1.03E-16
TERG_02861	40S ribosomal protein S23	-1	1.43E-46
TERG_06844	40S ribosomal protein S21	-1.2	7.06E-13
TERG_03057	Uncharacterized protein	-1.8	1.85E-33
TERG_05345	60S ribosomal protein L43	-1	1.86E-37
TERG_06158	60S ribosomal protein L29	-1.2	8.70E-35
TERG_06824	60S ribosomal protein L23	-1	3.28E-33
TERG_11688	60S ribosomal protein L31	-1.2	1.66E-26
TERG_03669	54S ribosomal protein L31, mitochondrial	-1.5	5.55E-05
**Ribosome biogenesis**			
TERG_04062	Low-temperature viability protein ltv1	-1	1.51E-04
TERG_04746	Nucleolar GTP-binding protein 1	-1	1.02E-16

TERG_08497	Ribosome assembly factor mrt4	-1	3.72E-05
TERG_04441	H/ACA ribonucleoprotein complex subunit CBF5	-1.1	1.95E-14
TERG_04221	H/ACA ribonucleoprotein complex subunit NOP10	-2.1	2.72E-34
TERG_12452	Nucleolar GTP-binding protein 2	-1.2	6.83E-06
TERG_05388	Histone-binding protein RBBP4 N-terminal domain-containing protein	-1	2.05E-09
TERG_00604	Nucleolar protein 16	-1	1.19E-06
TERG_05368	60S ribosome subunit biogenesis protein NIP7	-1.1	1.86E-03
**Transport proteins**			
TERG_01820	Major facilitator superfamily (MFS) profile domain-containing protein	-1	2.56E-08
TERG_02508	ABC multidrug transporter MDR1 (Multidrug resistance protein 1)	-1.3	1.22E-46
TERG_08336	MFS-type efflux pump MFS2	-1.3	1.14E-149
TERG_05248	Major facilitator superfamily (MFS) profile domain-containing protein	-1.1	2.84E-11
TERG_05209	Mitochondrial outer membrane translocase complex, subunit Tom5	-1	3.31E-07
TERG_06788	Zinc/iron transporter	-1.1	2.11E-51
TERG_07923	Amino acid transporter transmembrane domain-containing protein	-1.3	4.65E-65
TERG_12018	Major facilitator superfamily (MFS) profile domain-containing protein	-1.1	6.98E-12
**Lyase activity**			
TERG_03256	Alpha-carbonic anhydrase domain-containing protein	-1.2	2.77E-03
TERG_04917	Pyruvate decarboxylase (EC 4.1.1.1)	-1.1	2.13E-07
TERG_04924	Aromatic amino acid beta-eliminating lyase/threonine aldolase domain-containing protein	-1.1	8.97E-05
TERG_06675	gamma-glutamylcyclotransferase (EC 4.3.2.9)	-1.5	3.90E-41
TERG_11637	methylisocitrate lyase (EC 4.1.3.30)	-1.4	1.20E-16
TERG_03256	Alpha-carbonic anhydrase domain-containing protein	-1.2	2.77E-03
**Oxidoreductase activity**			
TERG_00823	Short chain dehydrogenase/reductase	-1.2	2.23E-26
TERG_01858	S-(hydroxymethyl)glutathione dehydrogenase (EC 1.1.1.284)	-1.1	5.20E-21
TERG_02031	Methylenetetrahydrofolate dehydrogenase	-1.3	1.08E-21
TERG_02747	Cytochrome P450 alkane hydroxylase	-1.9	2.73E-128
TERG_03078	Bifunctional cytochrome P450/NADPH--P450 reductase	-1	1.39E-89

TERG_03305	Fatty acid hydroxylase domain-containing protein	-1	2.78E-02
TERG_03617	Monooxygenase	-1	1.21E-21
TERG_04382	C-14 sterol reductase	-1.1	5.24E-33
TERG_07584	Short chain dehydrogenase/reductase	-1.4	9.95E-08
TERG_08355	Enoyl reductase (ER) domain-containing protein	-1	1.26E-08
TERG_12299	Uncharacterized protein	-1.5	7.10E-05
TERG_12300	Cytochrome P450 monooxygenase	-1.3	4.47E-06
TERG_02745	Phosphoglycerate dehydrogenase	-1.1	2.34E-09
TERG_01180	FAD-binding domain-containing protein	-1.4	3.03E-23
TERG_06757	Thioredoxin	-1.4	7.47E-18
**Hydrolases**			
TERG_11593	Carboxypeptidase	-1	2.02E-09
TERG_03552	Carboxypeptidase (EC 3.4.16.-)	-1.4	2.54E-05
TERG_06622	Caspase family p10 domain-containing protein	-1.1	9.43E-31
TERG_04340	AB hydrolase-1 domain-containing protein	-1.1	4.65E-10
TERG_01907	AB hydrolase-1 domain-containing protein	-1.1	4.15E-04
TERG_05854	Beta-lactamase-related domain-containing protein	-1.1	6.59E-11
TERG_03382	DJ-1/PfpI domain-containing protein	-1.3	3.23E-95
TERG_08048	NAD dependent epimerase/dehydratase	-1	2.67E-05
**Ligases**			
TERG_00775	AMP-dependent synthetase/ligase domain-containing protein	-1.2	4.25E-27
TERG_06221	CTP synthase (EC 6.3.4.2) (UTP--ammonia ligase)	-1.4	4.40E-18
TERG_06421	Ubiquitin-related modifier 1	-1.7	2.21E-02
**Transferases**			
TERG_07734	O-methyltransferase	-1.4	2.91E-72
TERG_02528	Arsenite methyltransferase (EC 2.1.1.137)	-1.1	9.19E-65
TERG_07408	N-acetyltransferase domain-containing protein	-1	9.90E-12
TERG_05545	N-acetyltransferase domain-containing protein	-1.6	6.81E-14
TERG_02317	Phosphatidylethanolamine N-methyltransferase (PE methyltransferase)	-1.1	9.61E-05
TERG_01902	Aminoglycoside phosphotransferase domain-containing protein	-1	2.12E-03
TERG_04960	Glutathione transferase	-1	2.77E-15
TERG_00579	GST N-terminal domain-containing protein	-1.1	5.90E-05
TERG_04303	GST C-terminal domain-containing protein	-1	8.66E-19
TERG_05883	Squalene synthase (SQS) (SS) (EC 2.5.1.21)	-1.8	1.80E-05
TERG_06091	DNA-directed RNA polymerase I	-1	5.20E-09
TERG_02619	CobW C-terminal domain-containing protein	-2.3	1.50E-13

TERG_03751	Phosphoglycerate mutase	-1.3	3.98E-22
TERG_02147	PhnB-like domain-containing protein	-1.2	7.32E-05
TERG_06533	VOC domain-containing protein	-1.3	1.68E-09
TERG_01669	Methyltransferase	-1.1	6.05E-06
**Regulatory proteins**			
TERG_01075	C2H2-type domain-containing protein	-1.1	2.71E-02
TERG_01956	C2H2-type domain-containing protein	-1	4.34E-17
TERG_02529	BZIP domain-containing protein	-1.1	3.02E-17
TERG_06055	NmrA-like domain-containing protein	-1.3	5.22E-11
TERG_04247	CMGC/SRPK protein kinase	-2.2	2.22E-13
TERG_00854	NmrA-like domain-containing protein	-1.6	1.84E-05
**Others/hypothetical**			
TERG_02610	YjgH family protein	-1.2	4.37E-25
TERG_03789	Glutamine-serine rich protein MS8	-1.1	1.31E-08
TERG_00844	DNA recombination and repair protein Rad51-like C-terminal domain-containing protein	-1	2.07E-03
TERG_05617	G domain-containing protein	-1.5	3.00E-08
TERG_06804	EKC/KEOPS complex subunit CGI121 (EKC/KEOPS complex subunit cgi121)	-1	8.93E-09
TERG_07698	CHL4 family chromosome segregation protein	-1.1	1.20E-14
TERG_08422	Proteinase inhibitor I78	-1.4	1.51E-81
TERG_08277	E3 ubiquitin ligase complex SCF subunit sconC (Elongin-C)	-1.3	5.54E-03
TERG_01566	INSIG domain-containing protein	-1.3	2.42E-04
TERG_03252	Uncharacterized protein	-1	1.37E-08
TERG_11655	Uncharacterized protein	-1.2	4.14E-04
TERG_11701	Uncharacterized protein	-1.1	2.55E-02
TERG_11771	Uncharacterized protein	-1.6	1.39E-04
TERG_11946	Uncharacterized protein	-1.9	6.46E-27
TERG_12032	Uncharacterized protein	-1.7	1.28E-15
TERG_12382	Uncharacterized protein	-1	2.71E-10
TERG_12526	Uncharacterized protein	-1.7	7.65E-10
TERG_12533	Uncharacterized protein	-1	1.11E-13
TERG_12669	Uncharacterized protein	-1.5	3.72E-11
TERG_01218	Uncharacterized protein	-1	2.27E-06
TERG_01399	Uncharacterized protein	-1.5	5.19E-09
TERG_01527	Uncharacterized protein	-1.5	3.60E-06
TERG_02902	Uncharacterized protein	-1	9.09E-52
TERG_00523	Uncharacterized protein	-1.6	9.20E-47
TERG_00598	Uncharacterized protein	-1	1.49E-06
TERG_08503	Uncharacterized protein	-1.4	6.79E-21
TERG_08918	Uncharacterized protein	-1.2	1.09E-05
TERG_11559	Integral membrane protein	-1.2	3.07E-04
TERG_07830	Uncharacterized protein	-1.6	1.33E-20

TERG_05818	Uncharacterized protein	-1.1	8.85E-07
TERG_07094	Uncharacterized protein	-1.2	1.49E-22
TERG_07351	Uncharacterized protein	-1.2	1.32E-23
TERG_02746	Uncharacterized protein	-1.9	1.42E-17
TERG_01768	Uncharacterized protein	-1.3	2.19E-03
TERG_00967	Uncharacterized protein	-3.2	2.52E-96

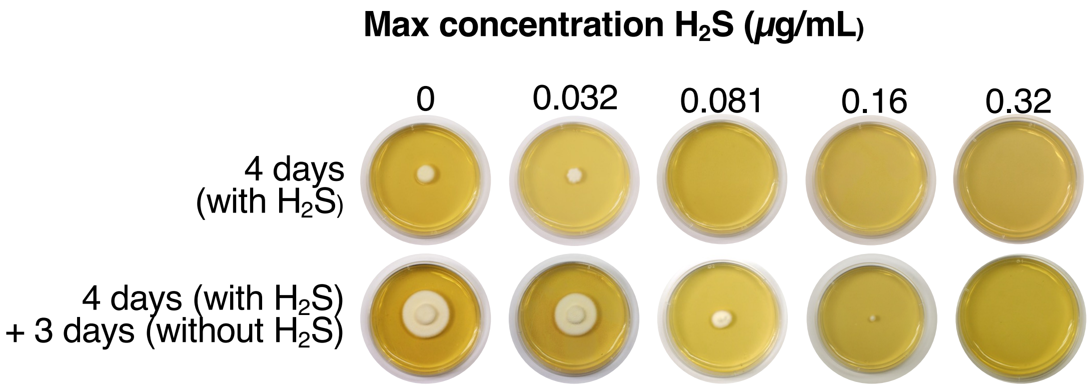

Fig S1. Minimal inhibitory and minimal fungicidal concentration of H_2S against *T. rubrum* ATCC 28188. The top row shows *T. rubrum* growth in the presence of H_2S, in an airtight box, for 4 days, to determine the MIC_{g_max}. After this, the plates were taken out of the box and incubated for a further 3 days to determine the MFC_{g_max} (bottom row).
